# Developing Lignosulfonate-Based Activated Carbon Fibers

**DOI:** 10.3390/ma11101877

**Published:** 2018-10-01

**Authors:** Feng-Cheng Chang, Shih-Hsuan Yen, Szu-Han Wang

**Affiliations:** 1 Advanced Research Center for Green Materials Science and Technology, National Taiwan University, #1, Sec. 4, Roosevelt Rd., Taipei 10617, Taiwan; r06625006@ntu.edu.tw; 2 School of Forestry and Resource Conservation, National Taiwan University, #1, Sec. 4, Roosevelt Rd., Taipei 10617, Taiwan; r02625035@ntu.edu.tw

**Keywords:** activated carbon fiber, carbonization, electrospinning, lignosulfonate, specific surface area

## Abstract

In this study, electrospinning technology, physical activation, and carbonization processing were applied to produce lignosulfonate-based activated carbon fibers. The porous structure of the produced lignosulfonate-based activated carbon fibers primarily contained mesopores and a relatively small amount of micropores. Moreover, insufficient carbonization caused fiber damage during CO_2_ activation. The weight loss rate and specific surface area increased with increase in carbonization time, and products with carbonization temperatures of 700 °C were of higher quality than those with other temperatures. Moreover, the two-step carbonization process provided fibers with improved quality because of a low weight loss rate, improved processing, and high surface area. Lignosulfonate-based activated carbon fibers can be used as a highly efficient adsorption and filtration material, and further development of its applications would be valuable.

## 1. Introduction

Activated carbon is a carbon material that comprises numerous mesoporous and microporous structures. Because of excellent adsorption, absorption, hydrophilicity, and specific surface area, activated carbon is generally used as an adsorbent in food and environment applications. The porous structure of activated carbon can physically or chemically interact with the adsorbed subjects to eliminate colored, odorous, or harmful substances. Because of its effective function of adsorbing and eliminating impurities and pollutants, activated carbon is being widely used in food, medical, solvent recovery, drinking water treatment, waste gas, wastewater treatment, and fuel cell industries [1].

Raw materials such as coconut shells, rice husks, bamboo, coal, asphalt, phenolic, polyacrylonitrile (PAN), cellulose, and polymer resins are generally used as active carbon precursors, among which PAN is the most widely used. The properties of the carbons produced varies depending on the precursor material [2]. Activated carbon processing, which varies according to the raw material, typically includes three stages: preoxidation, carbonization, and activation. The primary objective of activation treatment—which can generally be categorized as physical activation and chemical activation—is to increase the porosity and surface area of carbon materials in order to improve the adsorption capacity. In physical activation, CO_2_ and water steam are used as the activation agent [3], while H_3_PO_4_, ZnCl_2_, H_2_SO_4_, KOH, and K_2_CO_3_ are some common chemical activation agents.

According to Rodríguez-Reinoso [4], during pyrolysis, heteroatoms, such as O, H, and N, form gaseous products and are eliminated as volatile gaseous products prior to activation. Residual carbon elements randomly form groups and stacks between flat aromatic sheets, and gaps develop between sheet laminates because of irregularities. These laminates may be filled with or partially blocked by tars and other decomposition products that become disorganized carbons. These disorganized carbons first react with gases in the activation process and empty the pores, thus improving the surface area as well as the adsorbent capacity.

Depending on shape and use, activated carbon can be categorized into three types: powder activated carbon (PAC), granular activated carbon (GAC), and fibrous activated carbon or activated carbon fiber (ACF) [1]. PAC is obtained by carbonizing and activating materials and grounding and sieving them to form fine powders of diameter less than 0.043 mm. Because of its high specific surface area and strong adsorption capacity, PAC can be used as adsorbent material in numerous applications. However, PAC may cause dust problems given its fine particle size. GAC can be of many shapes, for example, cylindrical, spherical, and crushed random shapes. Compared with PAC, GAC has a smaller surface area and inferior adsorption capacity. However, because of the relatively large particle size of GAC, device clogging is less likely. GAC can be easily regenerated and thus is the most widely used activated carbon.

ACF is typically prepared by activating carbon fibers produced using common precursors, such as PAN, rayon, and pitch. ACF has favorable advantages, such as large surface area, uniform pore size, strong adsorption ability, ease of regeneration, and satisfactory mechanical properties. However, ACF is relatively expensive, and the amount of adsorption is relatively low [1]. Given the increasing severity of environmental pollution, highly efficient activated carbon materials are currently receiving considerable research attention. In terms of the efficiency of filtration materials, ACF with an ultrafine porous structure may be useful because of its high surface area–induced strong absorption ability and the complex network structure. Compared with granular and PAC materials, ACF is favorable for a variety of applications, such as making electrode for electrochemical devices [5,6].

Lignin, an amorphous polymer, is one of the three primary chemical components of the lignocellulosic biomass cell wall, which forms 15–40% of the dry mass of wood; it acts as a structural component and as conducting tissues in vascular plants and occupies 20–30% of terrestrial biomass [7]. However, conventionally, in the pulp and paper industry, chemicals are applied to eliminate lignin during pulping, and technical lignin are produced as byproducts; these are generally discarded or used as a fuel source, whereas a very small quantity is used in commercial applications [8,9,10]. The available technical lignin is not used efficiently, which means waste of resources.

The use of technical lignin as precursors to produce carbon fibers has been extensively studied [11,12,13,14,15,16,17,18,19]. In particular, submicron-scale lignosulfonate-based carbon fibers were fabricated through a series of electrospinning and carbonization processes [15,18,19]. After carbonization, an activation process can be implemented to generate submicron- or nanoscale lignin-based activated carbon fibers with small diameters and large surface areas, which could have potential in many applications (e.g., filtration and electrochemical properties). However, fibrous lignin-based activated carbon was relatively less studied in previous research since lignin-based activated carbon materials were commonly studied as granular and powder [19,20,21,22], not many as a fine fibrous format. Hu and Hsieh’s work [23] was one of the few studies about fine structure lignin-based activated carbon fibers, using electrospun alkali lignin fibers treated with chemical activation. Therefore, the process of fibrous lignin-based activated carbon fiber and its practical applications need to be further studied to avoid the wastage of natural resources, and thus this development contributes to environmental preservation efforts.

In light of technological advancement, the development and application of high-performance, porous adsorption materials of small size and high specific surface area are crucial for future development. Therefore, this preliminary study was conducted to investigate the feasibility of producing submicron-scale lignosulfonate-based ACFs (LACFs). Lignosulfonate fibers were fabricated through electrospinning technology for use as fibrous precursor, following which it was subjected to carbonization and physical activation. The influence of processing conditions on various properties of the products was studied.

## 2. Materials and Methods

### 2.1. Materials

The precursor of the carbon fiber was prepared by dissolving soluble lignosulfonates (hardwood lignosulfonic acid sodium salt (HLS), Borregaard, Sarpsborg, Norway; M*w* = 8000 g/mol) in reverse-osmosis water. To facilitate electrospinning, a small portion of poly(ethylene oxide) (PEO, Acros, Livingston, NJ, USA; M*w* = 600,000 g/mol) was dissolved in an HLS solution. The solution was mixed and heated in an oil bath at 80 °C and vortexed until it was completely dissolved; it allowed for cooling to room temperature before electrospinning. The lignosulfonate solution used was 20 wt.% mixture, containing 97 wt.% lignosulfonates and 3% PEO, as described in the literature [15]. All of the chemicals were used as received.

### 2.2. Methods

#### 2.2.1. Electrospinning

**Figure 1:**
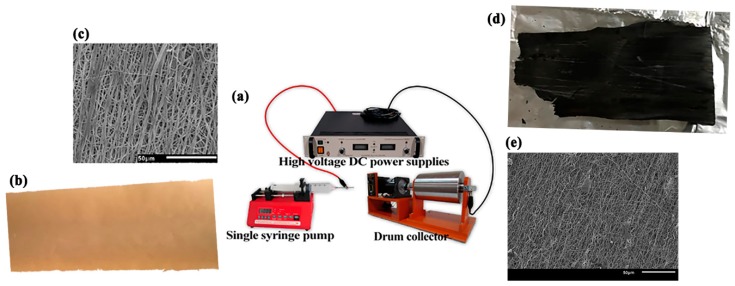
**(a)** electrospinning apparatus; (**b**) fabricated lignosulfonate fibers; (**c**) fabricated lignosulfonate fibers’ SEM; (**d**) activated carbon fibers; (**e**) activated carbon fibers’ SEM.

Electrospinning was performed in the horizontal direction (Figure 1a). For electrospinning, the formulated HLS solution was loaded in a syringe and then charged using a power supply (EL50P0, Glassman High Voltage Inc., High Bridge, NJ, USA). The syringe needle and a collector were connected to the positive terminal and ground of the power supply, respectively, and the applied voltage was 15 kV. The flow rate, syringe-to-collector distance, collector rotating rate, and needle gauge were 0.03 mL/min, 20 cm, 720 rpm, and 18 G, respectively. The electrospun HLS fibers were collected on a substrate to form lignin fiber mats. The average thickness of fiber mats and diameter of the fibers were 43.70 ± 3.09 μm and approximately 1611 ± 351 nm, respectively (Figure 1b,c). The final LACF product and its scanning electron microscopic (SEM) images were also shown in Figure 1d,e, respectively.

#### 2.2.2. Production of Activated Carbon Fibers

In this study, LACFs were fabricated in two stages: preoxidation and carbonization. As described in Yen and Chang [15], the electrospun lignosulfonate fiber mat was heated to 250 °C at the rate 1 °C/min under air environment, kept isothermal for 1 h, and cooled gradually to ambient temperature. Afterwards, the pre-oxidized lignosulfonate fiber mat was carbonized using a tube furnace (Barnstead Thermolyne F59300, Conroe, TX, USA) under N_2_ environment. Moreover, the carbonization was performed in two stages. In the first stage, the pre-oxidized fiber was treated at 400 °C for 5 min at a heating rate of 1 °C/min. In the second stage, the fiber was treated at a higher temperature for 1 h at a heating rate of 12 °C/min, and the temperature used was 400, 500, 600, and 700 °C.

**Figure 2:**
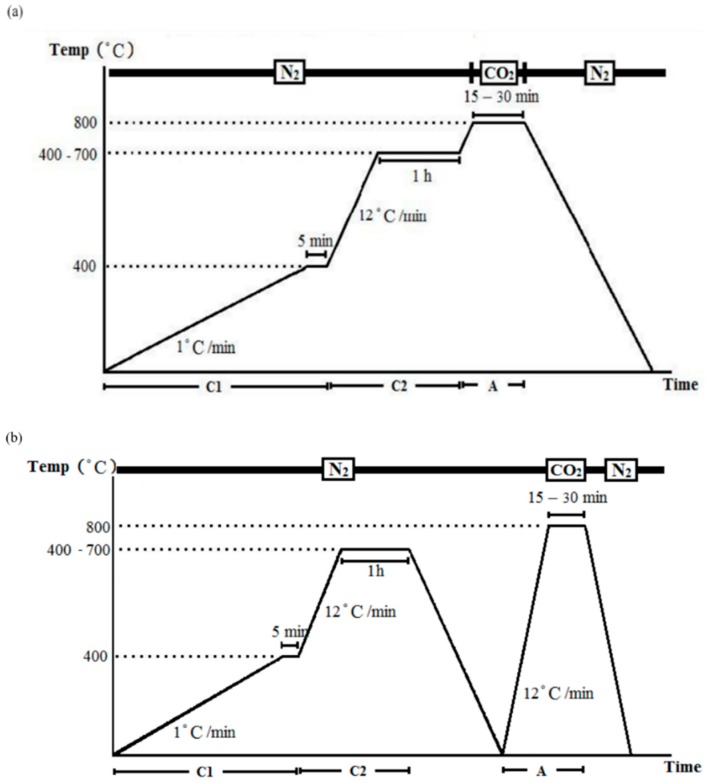
Carbonization and activation: (**a**) one-step process and (**b**) two-step process.

To avoid the use of chemical solvents, physical activation was employed. According to Hernández–Montoya et al. [24], compared with steam, the use of CO_2_ as the activation agent at the same temperature produces fibers with small pore size, large pore volume, and low weight loss of fibers. Therefore, CO_2_ was used as the activation agent. Furthermore, Baklanova et al. [20] indicated that a carbonization temperature of 700 °C provides a narrow pore diameter, which increases with increase in the carbonization temperature. In addition, Yun et al. [25] mentioned that the porous structure broadens when the activation temperature exceeds 800 °C due to damage to the mesoporous structure and that the produced materials at 800 °C have a high specific surface area [25]. In this study, several carbonization temperatures were used, including 400, 500, 600, and 700 °C and activation temperature was fixed at 800 °C. The specimen was activated in the same tube furnace using two activation methods:

1. One-step activation: the temperature increased to 800 °C after carbonization without cooling, and was maintained isothermally at 800 °C for 15 or 30 min in a CO_2_ environment, and then cooled to the ambient temperature in an N_2_ environment (Figure 2a).
2. Two-step activation: a cooling procedure was conducted between carbonization and activation, in which the temperature was increased to 800 °C in N_2_ environment, and then kept in an isothermal CO_2_ environment (800 °C) for 15 or 30 min before finally cooled to ambient temperature in an N_2_ environment (Figure 2b).

Weight loss during activation was determined using the following formula:

(1) $$\mathrm{Weight}\;\mathrm{loss}\;\left(\%\right)\;=\;[\left(W_{0}-W_{1}\right)/W_{0}]\times 100,$$

where *W*_0_ = original weight of fiber mat (g) and *W*_1_ = activated carbon fiber weight (g).

#### 2.2.3. Properties of Produced Activated Carbon Fibers

A scanning electron microscope (SEM, JEOL JSM–5410, Tokyo, Japan) was used to optically characterize the fiber surface structure and to measure the diameters of groups of 50 fibers. Different groups were compared through analysis of variance (ANOVA, α = 0.05) to determine the effects of the processing conditions. A Tukey test (confidence level, 95%) was performed to evaluate differences among the test groups.

Specific surface area and pore-size distribution are two common indices used to evaluate activated carbon materials; these properties were measured using gas adsorption–based surface area and porosimetry analyzers (ASAP 2010, Micromeritics, Norcross, GA, USA and NOVA touch^TM^, Quantachrome Instruments, Boynton Beach, FL, USA). Nitrogen was used in the degas treatment and as analytical gas. Liquid nitrogen was used as condensate liquid. P_0_ is the saturated vapor pressure of gas at liquid-nitrogen temperature (77 K). The adsorption type can be determined according to the isothermal adsorption curve, and the adsorption model suitable for the adsorbent can be determined. Previous studies have shown that the quenched solid density functional theory (QSDFT) can be applied to analyze pore-size distribution in microporous and mesoporous materials [26,27]. The analysis of the surface area and pore-size distribution reported herein is based on the QSDFT model.

## 3. Results and Discussion

**Figure 3:**
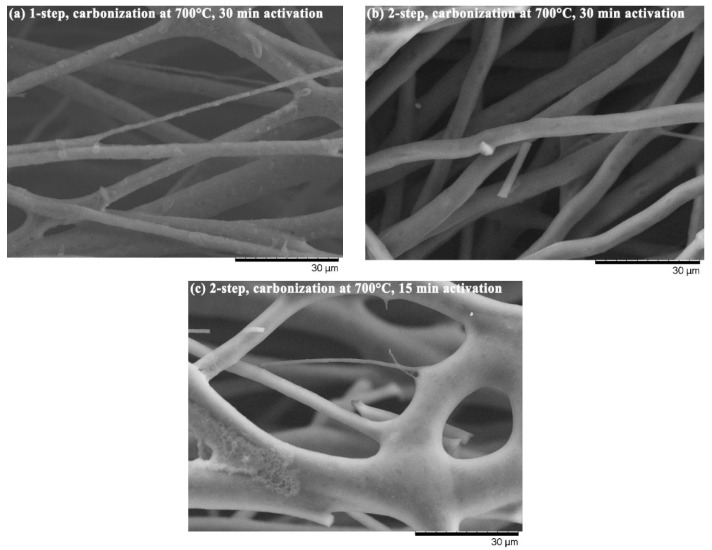
LACFs from different treatment conditions. (**a**) carbonization at 700 °C through one-step activation with activation time of 30 min; (**b**) carbonization at 700 °C through two-step activation with activation time of 30 min; (**c**) carbonization at 700 °C through two-step activation with activation time of 15 min.

Figure 3 indicates two main effects different procedures have on fiber morphology. First, the produced LACF has a rough surface when the activation treatment is short (c), whereas a smooth surface develops when the treatment duration is long (b). Second, the two-step process yields a smoother surface than does the one-step process (a). Pyrolysis byproducts, such as tar, could be observed on the LACF surface, and activation treatment eliminated these byproducts, leaving pores on the fiber surfaces. Based on the SEM image, it can also be observed that fibers may fuse during the processing, which results in larger fiber diameter. As for the specific area and pore structure of LACFs, they are influenced by carbonization and activation treatment conditions, which are discussed in the following sections.

Weight loss is one of the crucial referencing factors in the preparation of ACF; it can be used to determine the appropriate activation temperature. The experimental results (Table 1) indicate that when the activation holding time increased from 15 min to 30 min, the weight loss evidently increased. With increasing activation time, the fiber mats underwent damages and more parts were burned into ashes. Moreover, the carbonization temperatures significantly influenced the weight loss, and products made using the two-step activation generally resulted in lower weight loss than that made with one-step activation, which are similar to the results of Yun et al. [25].

For one-step activation, fibers were continuously treated at high temperatures for a long time; therefore, the resultant products have higher weight loss and frequently undergo damages during the process, whereas, for two-step activation, carbon fibers have formed during the first stage of carbonization and then cooled down. The second temperature increase may not cause considerable weight loss, and the structure of the product was relatively strong at maintaining a relatively intact shape and was not easily damaged by pore-drilling [25]. However, when the second temperature increased up to a temperature much higher than the first carbonization temperature, fiber weight loss may take place again since the carbonization was incomplete in the first stage. Therefore, the lower carbonization temperature in stage one would result in higher fiber weight loss. In addition, during activation, when the content of noncarbon elements in the fiber was higher, the fiber was more susceptible to damage because of oxidation.

Based on Alcañiz-Monge et al. [28], the diameters of fibers activated with CO_2_ would not considerably change with the increasing weight loss rate. However, results of this present study (Table 2) indicate that the diameters of groups treated through one-step activation were approximately 800 nm with no significant effect from the carbonization temperature, whereas those of groups treated through two-step activation exhibited large fiber diameters with the increase in carbonization temperatures. In one-step activation, the temperature directly increased up to 800 °C for carbonization and was then treated with CO_2_-based activation. However, two-step activation requires a second increase in temperature to up to 800 °C in a nitrogen atmosphere after carbonization. In this phase, the carbon fibers have formed; however, when the second temperature increased up to a temperature much higher than the first carbonization temperature, fiber shrinkage may take place again during the second carbonization and activation. Therefore, the resultant diameters of fibers were relatively small for the groups treated with a lower carbonization temperature. If treated with a higher carbonization temperature, the second temperature increase may not considerably influence the fiber diameters. Consequently, the results of two-step activation indicate that a higher carbonization temperature (700 °C) corresponds with the less susceptibility of the fiber to damage during activation.

**Figure 4:**
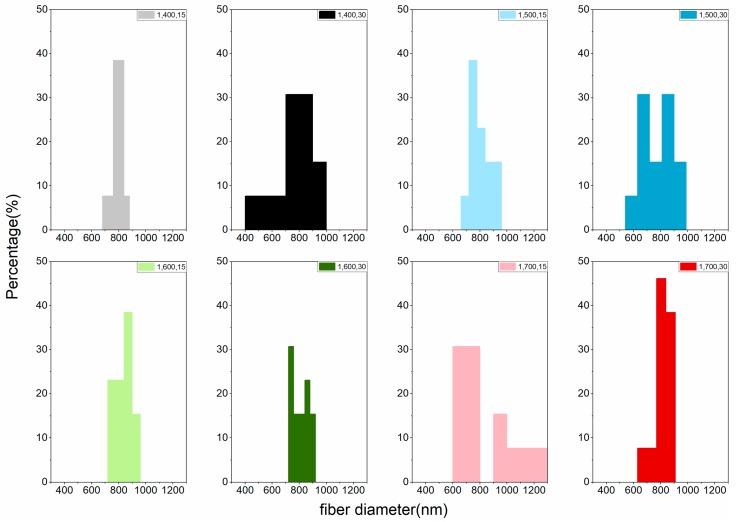
Fiber diameter distribution of the LACFs made with various one-step process, for each legend, 1: 1-step; 400–700: carbonization temperature (°C); 15, 30: activation time (min).

**Figure 5:**
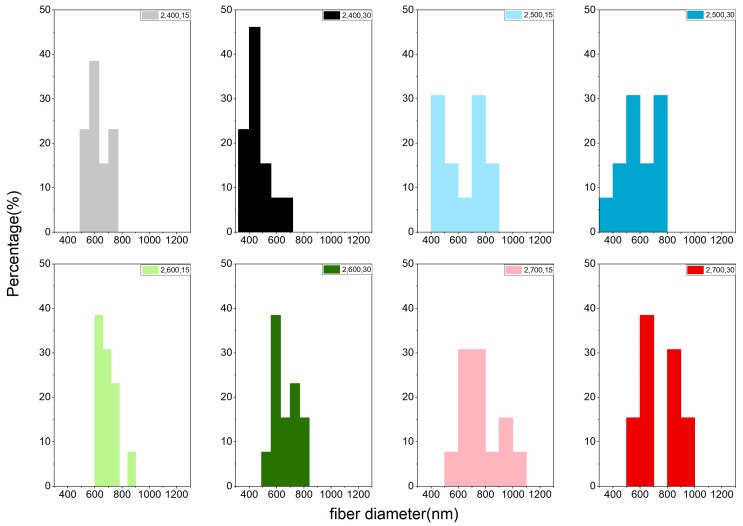
Fiber diameter distribution of the LACFs made with two-step process, for each legend, 2: 2-step; 400–700: carbonization temperature (°C); 15, 30: activation time (min).

The LACF fiber diameter distribution of each group can be found in Figure 4 and Figure 5. Variations of distributions among groups could be observed in the histogram and no apparent trend could be concluded to associate processing conditions to the diameter distribution. That may be attributed to the fusion of fibers during high-temperature processing [15,19], resulting in LACFs of large diameters and various distributions.

However, in terms of the average diameter of LACFs, carbonization temperature exerted a significant effect on fiber diameter, and increase in carbonization temperature increased fiber diameters for groups treated with the same heating programs. Moreover, no significant difference in the diameter was observed among the groups treated through one-step activation, whereas a significant difference in the diameter was observed among the groups treated through two-step carbonization (Table 2). Diameters of fibers activated using CO_2_ did not differ significantly from those of nonactivated fibers. Because CO_2_ has a high diffusion coefficient, fibers activated using CO_2_ produce deeper micropores without no change in fiber diameter. Hernández–Montoya et al. [24] mentioned a similar result.

**Figure 6:**
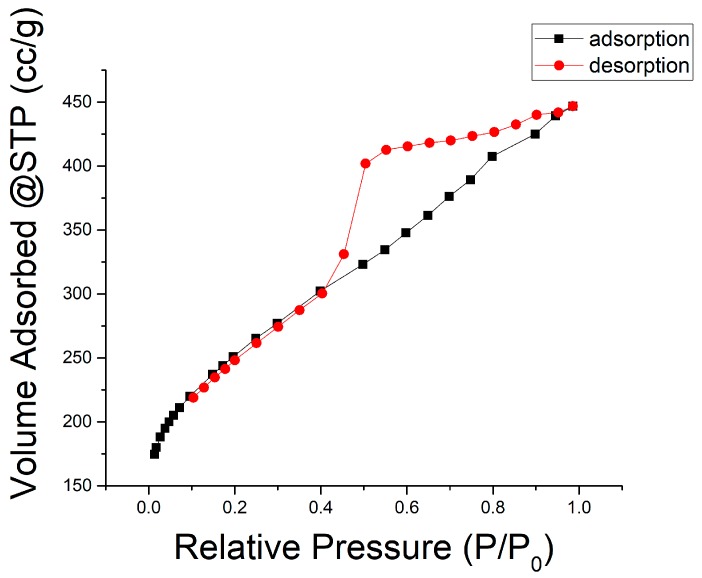
Nitrogen (77.35 K) physisorption isotherm (conditions: two-stage, 700 °C carbonization, 30 min activation).

Figure 6 is the typical nitrogen physisorption isotherm plot for the LACFs developed in this study. It is most similar to type IV(a) [29], although the knee part is not as bent as a typical IV(a) isotherm, and the type IV adsorption isotherms of lignin-based ACF were also reported in literature [23]. This indicates that the material mainly consists of mesopores and a relatively small amount of micropores. Based on the hysteresis loop shape, the lignin-based ACF can be categorized as the H2-b type. According to the International Union of Pure and Applied Chemistry (IUPAC) definition [29], the desorption branch falls abruptly when the relative pressure is approximately 0.30 because of pore blocking, which generally occurs within ink bottle-like pores. In this study, the bottleneck width is distributed over a wide range. Therefore, the pore structure of the activated carbon can be inferred to be ink-bottle pores connected by bottlenecks of various widths. Nevertheless, previously, the activated carbon fibers produced using rayon, pitch, and phenolic resins would have Langmuir-type adsorption isotherms [30,31,32].

**Figure 7:**
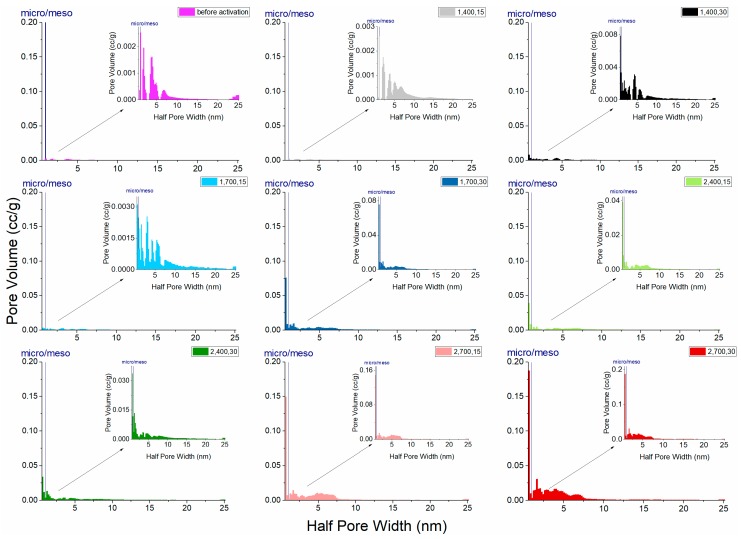
Pore-size distribution of the LACFs, for each legend, 1, 2: 1- or 2-step; 400, 700: carbonization temperature (°C); 15, 30: activation time (min).

Previous studies have demonstrated that activated carbon treated through the two-step process by using CO_2_ as the activation agent yields products with a high specific surface area, micropore volume, mesopore volume, and micropore proportion [24,25,33]. The development of porosity and active sites with a specific character is aided by physical activation because a partial oxidation occurs [24], as was the case in the experiment in this study (Figure 7 and Table 3). The results indicated that the material practically has mesopores in major and micropores in minor, according to overall observation. Figure 7 depicts the characteristic pore-size distribution in this study. Pore diameters in the LACFs ranged approximately from 1.6 to 48 nm, indicating that, aside from mesopores, micropores also occupy a slight part of the activated carbon, matching with the information observed based on adsorption isotherms, and the micropore proportion would vary with processing conditions.

In this study, the lignin fibers that were carbonized at 700 °C and activated at 800 °C for 30 min had a surface area of approximately 644 m^2^/g and pore volume 0.647 mL/g, indicating the potential to develop lignin-based activated carbon fibers for further applications. Although the values are not so high as other related studies that treated ACF with chemical activations [23], the results are comparable with another study that also used physical activation process [25], and higher than the results of PAC treated with chemical activation reported in one research paper [34].

Furthermore, Figure 7 and Table 3 indicate that, in one-step activation, high carbonization temperature or long activation time can provide high total pore volume and micropore proportion. In addition, in two-step activation, when carbonization temperature or activation time increase, the increased mesopores contribute to the pore volume and thus two-step activation generally resulted in higher pore volume than one-step activation. However, the pore volume is not changed much with a longer activation time, if the carbonization temperature is low.

Moreover, testing results (Table 3) indicate that, when carbonization temperatures were 400 °C, the specific surface area of the fibers after activation was lower than that of carbonized fibers without activation, implying that if carbonization is performed at a low temperature, drilling or pore expansion cannot be performed, and the specific surface area cannot be significantly increased even after activation.

## 4. Conclusions

Electrospinning technology was used to fabricate lignosulfonate fibers, which were carbonized and activated to produce LACFs. The effect processing conditions have on the products was discussed. The results showed that weight loss rate and specific surface area increased with increasing activation temperature. The optimal carbonization temperature in this study is 700 °C; the temperature of carbonization affects the activation of fibers, and incomplete carbonization can damage the fibers through activation.

Furthermore, carbonization temperatures have a significant effect on the diameters of LACFs when two-step activation was applied, and two-step activation treatment provides products with a low weight loss rate and high specific surface area. In addition, the weight loss increased with increasing activation time, and the two-step activation generally resulted in lower weight loss than one-step activation did. The results of two-step activation indicate that fibers treated with a higher carbonization temperature resulted in less susceptibility to damage during activation.

On the other hand, the LACFs produced in this study have a high specific surface area and primarily contain mesopores and a relatively small amount of micropores. The two-step process can produce LACFs with higher specific surface area and larger pore volume than does the one-step process, and the material practically has mesopores in major and micropores in minor.

Moreover, in two-step activation, when carbonization temperature or activation time increases, the increased mesopores contribute to the pore volume and the pore volume proportion, and the pore structure of the activated carbon can be inferred to be ink-bottle pores connected by bottlenecks of various widths. Consequently, lignosulfonate-based activated carbon fibers can be used as a highly efficient adsorption and filtration material, and further development of its applications would be valuable.

## Figures and Tables

**Table 1:** Weight loss of produced LACFs.

Carbonization Temperature (°C)	Weight Loss (wt.%)			
	One-Step Activation		Two-Step Activation	
	15 min	30 min	15 min	30 min
400	27.70 ^a^	38.62 ^a^	27.40 ^a^	50.01 ^a^
	(9.10)	(3.72)	(6.46)	(2.00)
500	25.37 ^ab^	44.89 ^b^	16.35 ^b^	41.96 ^b^
	(16.60)	(4.62)	(11.41)	(3.54)
600	18.58 ^bc^	32.34 ^c^	3.67 ^c^	31.43 ^c^
	(3.27)	(4.38)	(43.44)	(8.25)
700	11.92 ^c^	30.16 ^c^	3.99 ^c^	26.80 ^d^
	(16.16)	(3.48)	(57.70)	(4.65)

Numbers in parentheses are the coefficients of variation (%). In the same column, the same letter means no significant difference between groups.

**Table 2:** Diameter of produced LACFs.

Carbonization Temperature (°C)	Fiber Diameter (nm)			
	One-Step Activation		Two-Step Activation	
	15 min	30 min	15 min	30 min
400	792 ^a^	765 ^a^	622 ^a^	464 ^a^
	(5.67)	(18.09)	(13.94)	(22.56)
500	804 ^a^	794 ^a^	633 ^ab^	593 ^b^
	(9.93)	(13.61)	(26.11)	(22.63)
600	836 ^a^	807 ^a^	690 ^abc^	664 ^bc^
	(8.16)	(7.82)	(10.96)	(14.53)
700	841 ^a^	814 ^a^	760 ^bc^	746 ^c^
	(23.91)	(8.92)	(20.17)	(21.97)

Numbers in parentheses are the coefficients of variation (%). In the same column, the same letter means no significant difference between groups.

**Table 3:** Specific surface area of produced LACFs.

Treatment	Carbonization Temperature (°C)	Activation Time (min)	Surface Area (m^2^/g)	Pore Volume Proportion		Total Pore Volume (mL/g)
				Micro (%)	Meso (%)	
**Carbonization**	**700**		**112.15**	12.37	87.63	0.023081
One-Step Activation	400	15	64.10	9.50	90.50	0.028369
	400	30	23.43	20.11	79.89	0.059510
	700	15	147.40	13.18	86.82	0.047106
	700	30	327.29	39.53	60.47	0.218590
Two-Step Activation	400	15	98.43	35.04	64.96	0.136610
	400	30	112.42	25.96	74.04	0.183690
	700	15	369.94	35.17	64.83	0.448810
	700	30	643.89	30.79	69.21	0.646930
